# Multiobjective Gate Assignment Model Considering Carbon Emissions

**DOI:** 10.3390/ijerph20053952

**Published:** 2023-02-23

**Authors:** Yixuan Shan, Yuwei Shao, Qi Yuan, Yu Jiang

**Affiliations:** College of Civil Aviation, Nanjing University of Aeronautics and Astronautics, Nanjing 210016, China

**Keywords:** aviation, gate assignment, carbon mission, multiobjective model, NSGA-II

## Abstract

It has been a main concern for governments to reduce the carbon emission of the aviation industry. The paper proposes a multiobjective gate assignment model that considers the carbon emission at the airport surface to facilitate environmental-friendly airport construction. Three objectives are considered in the model to reduce carbon emissions, including the proportion of flights assigned to the contact gate, aircraft taxiing fuel consumption, and gate assignment robustness. In order to achieve better performance on all objectives, a Non-dominated Sorting Genetic Algorithm-II (NSGA-II) is used to obtain the optimal results. The operation data from a domestic airport is deployed to validate the model. The optimal results of the gate assignment model are compared with the original scheme. It indicates that the proposed model can effectively reduce carbon emissions. The study can provide a strategy for gate assignment to reduce carbon emissions and improve the management of the airport.

## 1. Introduction

In recent years, the environmental effect of civil aviation operations has become an important issue in air traffic management, especially for hub airports [1]. The flight congestion and delay resulting from the rapid growth of air traffic demand leads to a surge in carbon emissions. It has been reported that the carbon emissions of civil aviation transportation account for approximately 2% of the total carbon emissions of human activities [2]. It is necessary to find an efficient way for airports to operate more sustainably [3]. Therefore, how to reduce the carbon emissions of aircraft operations has become a crucial problem to solve.

The gate assignment of aircraft determines the taxiing distance and utilization of ground support equipment (GSE) at the airport surface, which are the main sources of aviation emissions [4,5]. Most existing studies focusing on gate assignments promote service quality, operation efficiency, or operation cost rather than decreasing carbon emissions [6,7,8,9,10]. To the best of the authors’ knowledge, the problem of gate assignment optimization with the aim of optimizing carbon emissions has yet to be adequately addressed. How to reduce taxiing carbon emissions and the extra emissions caused by conflicts at the surface when assigning gates to aircraft is the focus of this paper.

The contribution of this paper is the establishment of a multiobjective gate assignment model to maximize the proportion of flights assigned to the contact gate, minimize taxiing fuel consumption, maximize assignment robustness, and reduce the carbon emissions of operation. The gate assignment model can facilitate the establishment of environmentally friendly airports.

The remainder of the paper is structured as follows. A review of the literature related to gate assignment models and associated algorithms is presented in Section 2. The multiobjective gate assignment model considering carbon emissions and the solving algorithms are described in Section 3. In Section 4, the operation data of domestic airports are used to validate the model. Finally, the conclusions are highlighted in Section 5.

## 2. Literature Review

### 2.1. Gate Assignment Models

Many researchers have focused on the gate assignment problem. Most research has established single-objective models to optimize passenger walking distance or operation efficiency [11,12,13]. Some models integrate multiple objectives to optimize the gate assignment problem [6,8,14]. Single-objective optimization is further divided into passenger satisfaction, assignment plan robustness, airline benefits, etc. To improve airport capacity, Xiong et al. [11] developed a gate assignment model with the objective of minimizing the aircraft taxiing distance for multi-runway airports. Genc et al. [15] proposed minimizing the number of flights parked in remote boarding gates and solved the model using a composite algorithm. Wang et al. [12] proposed a single-objective model with the lowest cost of gate assignment. Li [13] built a model based on the actual business rules of the airport, with the maximum flight rate assigned to the contact gate as the optimization objective.

In recent years, more research has considered multiple interests simultaneously with multiobjective integrated optimization. Xue et al. [6] considered the fairness among airlines and established a multiobjective model for reducing the gate cost and taxi fuel consumption cost of airlines. Shen et al. [7] proposed establishing a gate pre-assignment model with the gate matching degree as the objective and integrating the near-airport flight-to-bridge ratio to carry out bi-objective optimization to maximize the gate utilization and travel satisfaction of passengers. Jiang et al. [8] constructed a multiobjective optimization model for gate assignment with the objectives of minimizing the probability of ramp conflicts, the walking distance of passengers for changing gates, and the number of passengers assigned to remote boarding gates. From a new perspective, Kumar et al. [16] considered three optimization objectives: minimum cost, maximum operating revenue, and robustness of airports, and proposed a multiobjective assignment model. Das [14] developed a multiobjective gate assignment model with the objectives of minimizing the travel distance by passengers and maximizing the airport stores passed by passengers on their route. From the perspective of airport resource use efficiency, Zheng et al. [17] constructed a bi-objective model with the optimization of maximizing the match between gate and aircraft type and minimizing the number of used gates.

In the field of air transport, due to the lack of technological breakthroughs in carbon-based fuel sources, a small number of scholars have begun to study ways to reduce carbon emissions from the perspective of airport operation management [18,19,20,21]. Sznajderman et al. [5] modeled several scenarios characterized according to gate assignment policy and analyzed different management model influences on the gaseous emissions generated by aircraft and ground support equipment altogether. Evertse [22] studied the problem of airport real-time taxiing path planning with the aim of minimizing emissions and used CPLEX software to solve the established model. Soltani et al. [23] introduced a MILP model to optimally control taxiing operations in an airport with an emphasis on conflict and tow-truck usage to minimize fuel consumption considering the concept of fixed parking positions. In summary, in terms of airport emission reduction management, the research is mainly focused on the study of aircraft taxiing [22,23,24,25,26], while there are few studies on gate assignment modeling considering multiple factors. Therefore, based on the theory of multiobjective programming, this paper established a gate assignment model and designed an algorithm to solve it for improved airport operation efficiency and reduced carbon emissions.

### 2.2. Algorithms

In recent years, domestic and foreign scholars have adopted various algorithms to solve different optimization objectives and constraints. Yan et al. [10] solved the joint optimal scheduling problem of flight taxiing path planning and parking space allocation in airports by an adaptive differential evolution algorithm but did not consider the flight landing rate and aircraft taxiing fuel consumption. Lu [27] restored the aircraft running in the scene through the NSGA-II algorithm but did not consider the taxiing fuel consumption, only the taxiing time. Based on the CART algorithm, Tang et al. [28] compared and analyzed the prediction accuracy of apron configuration characteristic variables before and after introduction in predicting the departure time of aircraft. However, the algorithm set taxiing as barrier-free taxiing without considering obstacles when taxiing. Li et al. [29] improved the genetic algorithm based on the structural characteristics of the flight area to reduce the fuel consumption of the aircraft in the airport flight area to achieve the lowest fuel consumption of the aircraft but did not consider the taxiing conflict time. Gök et al. [30] used a sum-element heuristic algorithm to solve the scheduling of airport turnover tasks and the route arrangement of the ground crew but did not include the conflict time at the intersection. Yin et al. [31] introduced a dictionary sequential optimization algorithm to allocate tarmac and runway for taxiing traffic in busy airports and allocated flights in sequence under the constraints of parking space availability and runway utilization but did not account for aircraft taxiing fuel consumption or the flight landing rate. According to the principle of priority, Wen et al. [32] introduced the gate and flight labeling function to design a labeling algorithm that can solve the scheduling model, which provided a feasible means for optimizing the automatic allocation of gates.

In summary, the optimization research on gate assignment mainly focuses on improving the heuristic intelligent optimization algorithm [29,33]. The research focuses on establishing the optimization goal of the gate assignment model, which leads to its simple constraints, low correlation with the actual airport business rules, and lack of practical guiding significance. Traditional genetic algorithms have the advantages of wide range, high robustness, and strong global search ability, but they easily fall into local solutions, and the convergence results are easy to repeat [34]. The nondominated sorting genetic algorithm (NSGA) has greatly improved speed due to layering individual relationships. In this paper, nondominated sorting genetic algorithm II (NSGA-II) [35], with the mutually exclusive characteristics of the three objectives in the model, is applied to optimize the gate assignment problem and obtain the nondominated set for multiobjective optimization problems.

## 3. Methodology

### 3.1. Multiobjective Gate Assignment Model

The gate assignment can directly influence the taxiing route of the flights. To be more specific, when the flight is assigned to the contact gate, it will reduce the taxiing distance of the flight, thus reducing carbon emissions. We propose increasing the proportion of flights assigned to the contact gate and reducing the taxiing fuel consumption of all flights to limit carbon emissions. In addition, the robustness of gate assignment will also impose an impact on carbon emissions. Increasing the gate assignment robustness can decrease the conflict caused by gate re-assignment and the waiting time of flights, which can benefit carbon emission optimization. Therefore, three objectives are considered when modeling the gate assignment: the proportion of flights assigned to the contact gate, the taxiing fuel consumption, and the gate assignment robustness. The reduction in carbon emissions when allocating the gates to flights can facilitate environmentally friendly airport construction. The objectives of the model are as follows:

(1) Improve the proportion of flights assigned to the contact gate

The proportion of flights assigned to the contact gate refers to the proportion of flights connecting with the terminal through the bridge. When the flight is assigned to a contact gate, the taxiing distance of the flight will significantly decrease, which can help reduce the carbon emissions of the flight. In addition, it has become a trend for bridges to be equipped with auxiliary power unit (APU) replacement facilities. The APU replacement facilities equipped in the gate are powered by electricity and thus do not generate carbon dioxide and other gases. Improving the proportion of flights assigned to contact gates can effectively reduce the carbon emissions of flights and improve the satisfaction of passengers because they can walk a shorter distance to board. The objective function (1) indicates the maximization of the proportion of flights assigned to the contact gate.

(1) $$Z_{1}=\max\underset{i\in F}{\sum }\underset{k\in G}{\sum }\frac{X_{ik}}{N}$$

where i is the number of flight pairs; F is the flight set; G is the gate set; $X_{ik}$ is the decision variable, which is equal to one if the flight i is assigned to the gate k and zero otherwise; and N is the number of flights.

(2) Reduce aircraft taxiing fuel consumption

The taxiing fuel consumption of flights is directly related to carbon emissions. Therefore, a decrease in taxiing fuel consumption can facilitate environmentally friendly airport construction. Moreover, it can also reduce the operation cost of airlines. In this paper, taxiing fuel consumption is calculated for the surface taxiing process by weighting the taxiing distance with the fuel flow rate when the aero-engine is idling-rated. The taxiing fuel consumption of flight pairs is proportional to the aircraft engine fuel flow rate, taxiing time and the number of engines [25]. The fuel consumption calculation formula of the flight pair is shown in Equation (2).

(2) $$F_{i}=\frac{d_{i}^{arr}+d_{i}^{dep}}{\bar{v}}\times n_{i}\times f_{i}$$

where $d_{i}^{arr}$ is the taxiing distance of the arrival flight; $d_{i}^{dep}$ is the taxiing distance of the departure flight; $\bar{v}$ is the average flight taxiing speed; $n_{i}$ is the number of engines; and $f_{i}$ is the engine fuel flow rate.

Therefore, the objective function (3) indicates the minimization of fuel consumption.

(3) $$Z_{2}=\min\underset{i\in F}{\sum }\underset{k\in G}{\sum }\left(\left(\frac{d_{k}^{arr}+d_{k}^{dep}}{\bar{v}}\right)X_{ik}\times n_{i}\times f_{i}\right)$$

(3) Increase gate assignment robustness

Increasing the gate assignment robustness considering the conflict time, can improve the utilization of gates, mitigate flight delays, and reduce the carbon emissions caused by congestion. Therefore, how to measure the robustness of gate assignments needs to be studied. The robustness of gate assignments refers to the anti-interference ability of the assignment scheme when a flight delay occurs, which can be measured by the expected conflict time. The smaller the expected conflict time is, the better the robustness performance of the gate assignment schedule. Therefore, this paper extracts the actual flight operation data of the airport to fit the flight delay distribution. The fitting function is used to estimate the expected conflict time [36], which is shown in Equation (4).

(4) $$T_{conflict}=52.4e^{-{\left(\frac{t_{i}^{arr}-t_{i}^{dep}-40.15}{51.91}\right)}^{2}}$$

where $t_{i}^{arr}$ is the arrival time of flight i, and $t_{i}^{dep}$ is the departure time of flight i.

The objective function (5) indicates the minimization of the conflict time (i.e., maximization of robustness):

(5) $$Z_{3}=\min\underset{i,j\in F}{\sum }\underset{k\in G}{\sum }X_{ik}\times X_{jk}\times Y_{ij}\times T_{conflict}$$

where $Y_{ij}$ is the decision variable, which is equal to one if flight i is consecutively followed by flight j in the same gate and zero otherwise.

The multiobjective gate assignment model is established to maximize the proportion of flights assigned to the contact gate, minimize the aircraft taxiing fuel consumption, and minimize the gate assignment robustness, which is shown in Equations (1), (3) and (5). The constraint sets are as follows:

(6) $$\underset{k\in G}{\sum }x_{ik}=1,\;\forall i,j\in F$$

(7) $$X_{ik}X_{jk}(t_{i}^{dep}-t_{j}^{arr})(t_{j}^{dep}-t_{i}^{arr})\leq 0,\forall i,j\in F;k\in G$$

(8) $$t_{j}^{arr}-t_{i}^{dep}+\left(1-Y_{ij}\right)M\geq T,\forall i,j\in F$$

(9) $$p_{i}\leq p_{k}+\left(1-X_{ik}\right)M,\forall i\in F;k\in G$$

(10) $$X_{ik},Y_{ij}\in \left\{0,1\right\}$$

where $p_{i}$ is the aircraft size; $p_{k}$ is the gate size; M is a large value; and T is the minimum time interval.

Equation (6) indicates that each flight must and can only be assigned to one gate; Equation (7) indicates that two flights cannot be assigned at the same gate at the same time; Equation (8) indicates that two flights on the same gate shall meet a certain safety time interval; and Equation (9) indicates that the gate type matches the aircraft type. Equation (10) indicates that $X_{ik},Y_{ij}$ are 0–1 variables.

### 3.2. Algorithm

**Figure 1:**
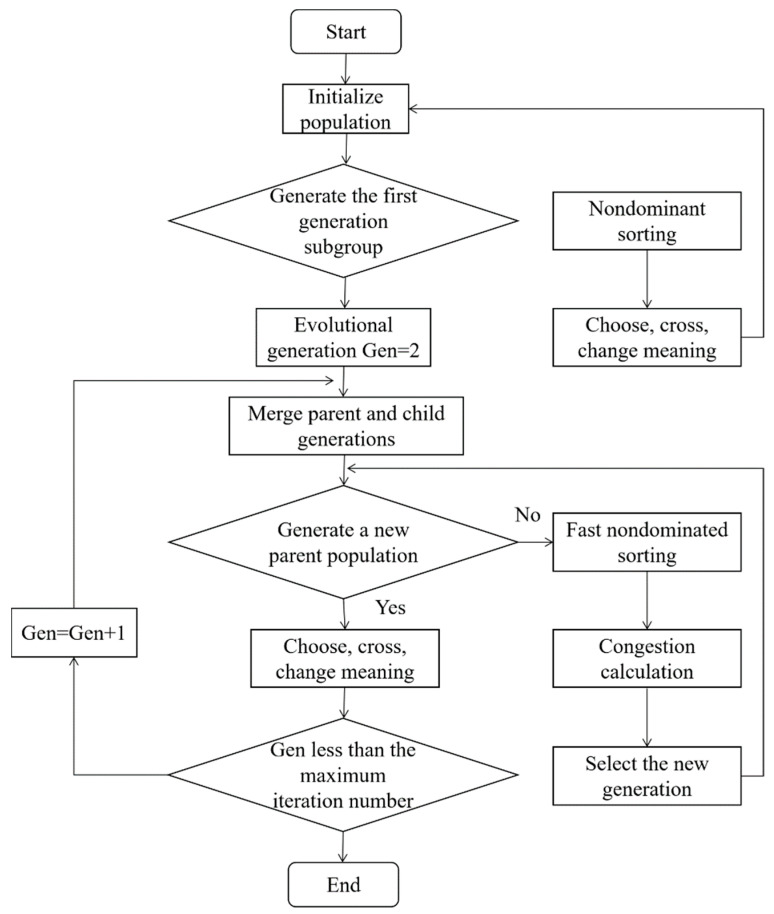
The basic process framework of the NSGA-II algorithm.

Non-dominated sorting genetic algorithm II (NSGA-II) is deployed to solve the multiobjective gate assignment model [35]. It refers to the process that classifies the population by nondominated sorting of the population, calculates the crowding distance of population individuals to maintain the diversity of the population, and obtains an approximate solution when the termination condition is reached. The basic process framework of the NSGA-II algorithm is shown in Figure 1.

The process of the NSGA-II is as follows:

Step 1: Code. The algorithm adopts decimal integer coding;

Step 2: Initialization. The algorithm initializes the population and generates the first-generation subgroup through nondominated sorting, choosing, crossing, and changing meaning;

Step 3: Generation of a new population. The algorithm merges the parent generation and the child generation to generate a new population;

Step 4: Determination of whether the termination conditions are met. If the end conditions are met, stop the program, and otherwise, repeat Step 2 to Step 3.

**Figure 2:**
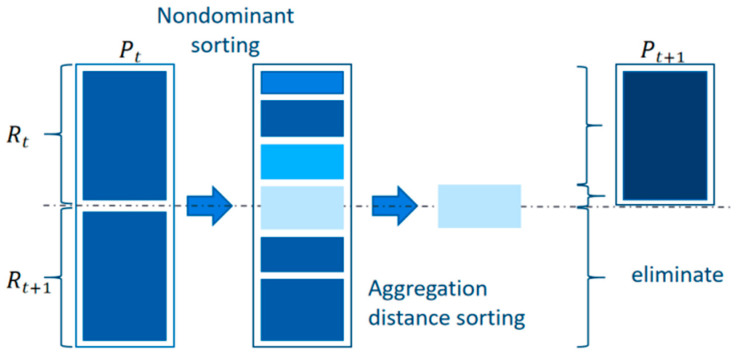
Example of a new species composition of the NSGA-II.

The algorithm adopts the decimal integer encoding method. The genes on chromosomes represent gate numbers, and the chromosome length is the total number of flights. An initial population with the scale of N is randomly generated as the first-generation parent population, according to the constraints used for the gate, to the extent feasible. The progeny population is then obtained by three basic operations: selection, crossover, and mutation. By starting from the second generation, the parent population and the progeny population are merged for fast nondominated sorting. At the same time, the crowding degree of individuals in each nondominated layer is calculated. According to the nondominated relationship and crowding degree of individuals, appropriate individuals are selected to form a new population. This process continues until the conditions for the end of the program are met. The composition of the new population of the algorithm is shown in Figure 2, where the dominance relationship refers to the fact that each objective function index of individual A is superior to that of another individual B, and thus A is dominant to B; otherwise, it is a nondominated relationship, which is divided into several layers according to the relationship between individuals. The individuals in the layer are nondominated, and the individuals in the upper layer are dominated by those in the lower layer.

## 4. Experimental Results

### 4.1. Example Introduction

**Figure 3:**
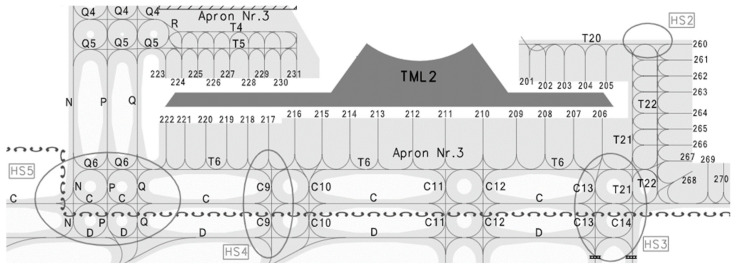
Gate location distribution of the airport terminals at ZSNJ.

In this paper, Nanjing Lukou International Airport (ZSNJ) was selected to validate the model. A total of 16 airport gates at the airport, including 13 contact gates (212–224) and 3 remote gates (266, 267, and 269), were selected to simulate the model. The configuration of the airport is shown in Figure 3. The areas marked HS on the map are conflict-prone areas of the airport. Information on each gate, including the gate number, type, and taxiing distance, is described in Table 1.

The flight operation data of the airport were also extracted. A total of 136 domestic flights taking off and landing at the airport from 8:30 to 18:00 on 1 September 2019, namely 68 domestic arrival and departure flights, were selected for simulation verification. The specific flight information is shown in Table 2. According to the aviation engine emission database released by the International Civil Aviation Organization (ICAO), the fuel flow rate of the engine in the idle state corresponding to each flight type can be obtained, as shown in Table 3.

### 4.2. Results and Discussion

The average taxiing speed of the aircraft was set as 15 km/h, and the shortest time interval of the same position was set as 15 min [6]. The maximum number of iterations was 200. This paper used the Visual Studio 2013 platform to achieve the NSGA-II algorithm.

**Figure 4:**
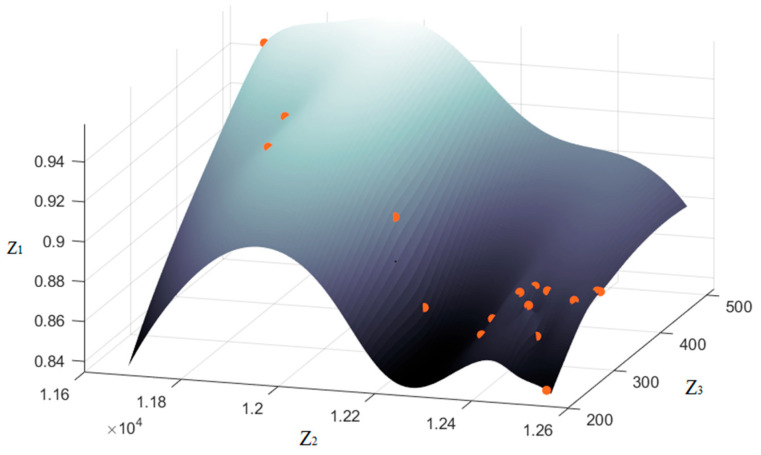
Pareto front solution.

A total of 17 groups of Pareto front solutions were obtained, and their distribution and fitting surfaces are shown in Figure 4. The Pareto front solutions are defined as nondominated solutions and have the least amount of model goal conflict, which provides decision-makers with a better choice space. It can be seen in Figure 4 that a more robust gate assignment plan will require more idle gate usage, which results in more aircraft being assigned to remote gates. It will decrease the proportion of flights assigned to contact gates and increase the total fuel consumption and carbon emissions. In the resource-constrained situation, the proportion of flights assigned to contact gates and gate assignment robustness present a negative relationship. Therefore, gate assignment needs to improve the utilization rate of the contact gate and reduce the overall carbon emissions while keeping the assignment robustness within a reasonable range.

The three best solutions were selected to be compared with the original scheme (OS) in Table 4. The OS of gate assignment is the original gate assignment provided by the airport. As seen in Table 4, the three assignment solutions do not dominate each other. Compared with the OS, the results of the gate assignment model have been improved for different objectives.

The first one is the result when Z_1_ and Z_2_ reach the optimum at the same time. The total taxiing fuel consumption and carbon emissions are significantly reduced compared with the actual assignment scheme, but the estimated total conflict time is 515 min, resulting in a high cost of flight delay and additional carbon emissions. Then, the result of optimal Z_3_ is presented. It has the lowest robust cost, but the remote gate rate and fuel consumption cost are too high. The result of optimizing the three objectives (Z_1_ + Z_2_ + Z_3_) is shown in Table 4. Compared with the original scheme, it increased the rate of flight assigned to the contact gate by 7.36% and reduced the total taxiing fuel consumption by 550.08 kg. Moreover, it reduced the total taxiing carbon emissions and fuel consumption by 2552.94 kg and 8.09 kg, respectively. However, it increased the estimated total conflict time by 50 min. In summary, the proposed gate assignment model can reduce carbon emissions to different degrees.

**Table 4:** Pareto front solutions based on the NSGA-II algorithm.

Objective(s)	Z_1_	Z_2_	Z_3_	Average Taxiing Fuel Consumption (kg)	Total Taxiing Carbon Emissions (kg)
OS	85.29%	12,401.44	284.42	182.37	57,555.16
Z_1_ + Z_2_	94.12%	11,670.32	515.66	171.62	54,162.01
Z_3_	83.82%	12,534.78	221.68	184.34	58,173.98
Z_1_ + Z_2_ + Z_3_	92.65%	11,851.36	334.57	174.28	55,002.22

**Figure 5:**
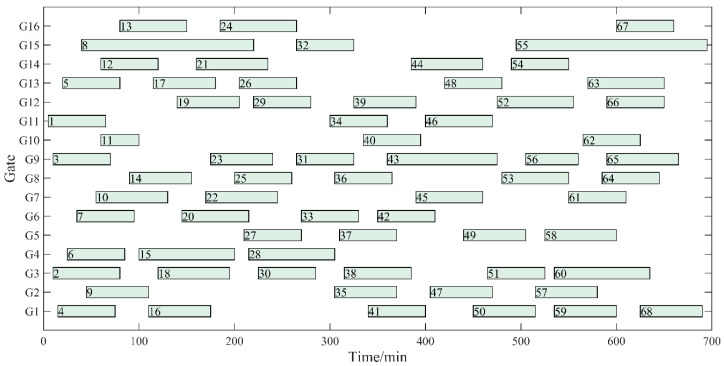
Gantt chart of the original gate assignment.

**Figure 6:**
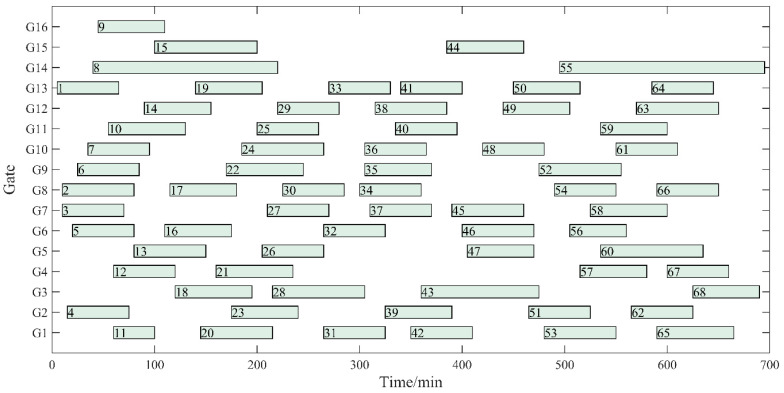
Gantt chart of the NSGA-II algorithm gate assignment solution.

A Gantt diagram of the original gate assignment is shown in Figure 5. The Gantt diagram of gate assignment with optimization of three objectives (Z_1_ + Z_2_ + Z_3_) is shown in Figure 6. As shown in Figure 5 and Figure 6, only five flight pairs with a long overpass time are assigned to the remote gates, which significantly improves the utilization efficiency of the contact gates. Since the model established in this paper fully considers the factors of taxiing distance and aircraft type, the mean and variance of fuel consumption are significantly reduced compared with the original assignment scheme, which indicates that the taxiing fuel consumption of each flight is relatively balanced. If we take Flight 18 as an example, the aircraft type is a medium-length wide-body aircraft, an A330-200. Its engine fuel flow rate is significantly higher than that of other small and medium aircraft, which greatly impacts the total carbon emissions. After the optimization in this paper, the flight was changed from remote gate 14 to contact gate 4, and the fuel consumption was reduced by 205.42 kg. The fuel-saving effect of a single flight was significant, and the overall fuel consumption was effectively reduced. Thus, carbon emissions are significantly reduced.

In summary, the simulation optimization result indicates that the proposed model can effectively reduce carbon emissions and simultaneously improve the utilization efficiency of contact gates, airport operation efficiency and robustness, thereby enhancing passenger satisfaction and reducing delay costs. In addition, the results show that the NSGA-Ⅱ algorithm can solve multiobjective models efficiently by fast non-dominated sorting and crowding sorting processes.

## 5. Conclusions

This paper proposes a multiobjective gate assignment model that considers carbon emissions to facilitate environmentally friendly airport construction. The proportion of flights assigned to the contact gate, aircraft taxiing fuel consumption, and gate assignment robustness are considered in the model to reduce carbon emissions. Multiple objectives of the gate assignment model interact with each other, which makes it challenging to explore the optimal solution. Therefore, in this research, the NSGA-II was used to solve the problem. Numerical studies have shown that the proposed gate assignment model could achieve better performance in decreasing carbon emissions compared with the original gate assignment plan. In addition, the proposed model can effectively reduce the walking distance of passengers, the operation cost of airlines, and the conflict time at the airport surface to different degrees. It has good market prospects for improving the economic benefits of airports and airlines while improving the utilization rate of airport gate resources.

Although the preliminary results are promising, there is still much research to be done. In this paper, only the partial process of gate assignment at the airport surface is considered, and the taxi route of the aircraft is assumed to be fixed. In addition, the mathematical models established in the current paper only considered deterministic cases, while several stochastic events, such as weather conditions, flight delays, and the impact of ground support operations on the apron, were not fully considered. Future research will focus on the joint scheduling of runway, taxiway and gate operations, considering multiple uncertainties to fully achieve the goals of energy conservation and emission reduction in airport operation.

## Figures and Tables

**Table 1:** Information on the gates of the ZSNJ airport.

Gate Number	Number	Gate Type	Gate Size	Arrival Taxiing Distance (m)	Departure Taxiing Distance (m)
212	G1	Near	3	1380	2450
213	G2	Near	3	1440	2390
214	G3	Near	3	1100	2330
215	G4	Near	3	1000	2275
216	G5	Near	3	920	2275
217	G6	Near	2	860	1870
218	G7	Near	2	920	1810
219	G8	Near	2	980	1780
220	G9	Near	2	1030	1730
221	G10	Near	2	1100	1670
222	G11	Near	2	1150	1580
223	G12	Near	2	1000	1150
224	G13	Near	2	1050	1320
266	G14	Far	3	1980	2880
267	G15	Far	3	1900	2950
269	G16	Far	3	2100	3000

Note: In the gate size column, 1 represents a small gate, 2 represents a medium gate, and 3 represents a large gate.

**Table 2:** Example of arrival and departure flight data at the ZSNJ airport.

Flight Pair Number	Arrival Flight Number	Arrival Time	Departure Flight Number	Departure Time	Aircraft Size	Engine	Engine Fuel Flow Rate (kg/s)
1	ZH9855	8:35	ZH9856	9:35	2	2	0.109
9	CZ6189	9:15	CZ6189	10:20	2	2	0.138
18	3U8923	10:30	3U8924	11:45	3	2	0.228
21	ZH9843	11:10	ZH9844	12:25	3	2	0.270
52	CZ6971	16:25	CZ6972	17:45	2	2	0.109

Note: In the Aircraft size column, 1 represents a small aircraft, 2 represents a medium aircraft, and 3 represents a large aircraft.

**Table 3:** Engine fuel flow rates under idle conditions.

Aircraft Type	Engine	Engine Type	Engine Fuel Flow Rate (kg/s)
32 N	2	LEAP-1A26CJ	0.091
A319	2	CFM56-5B5/P	0.102
A320	2	CFM56-5A3	0.104
A321	2	V2530-A5	0.138
A322	2	CFM56-5B4/P	0.102
A332	2	CF6-80C2B4	0.228
A333	2	Trent 772B-60	0.270
B737	2	CFM56-3C-1	0.124
B738	2	CFM56-7B24/2	0.109
B789	2	GEnx-1B70/72/P2	0.201

## Data Availability

No new data were created or analyzed in this study. Data sharing is not applicable to this article.
